# Arch-Shaped Triboelectric Nanogenerator Based on PTFE, Paper, and Aluminum for Sustainable Energy Harvesting

**DOI:** 10.3390/mi17091044

**Published:** 2026-09-01

**Authors:** Izhar Hussain, Kok Boon Ching, Shamsuddin Lakho, Safdar Ali Abro, Ghulam E Mustafa Abro, Sufyan Ali Memon, Muhammad Aslam

**Affiliations:** 1 Department of Electronics Engineering, Benazir Bhutto Shaheed University of Technology and Skill Development, Khairpur Mirs 66020, Pakistan; izharhussain@bbsutsd.edu.pk (I.H.); shamsuddinlakho@bbsutsd.edu.pk (S.L.); safdar@bbsutsd.edu.pk (S.A.A.); 2 Department of Electrical Engineering, Faculty of Electrical and Electronic Engineering, Universiti Tun Hussein Onn Malaysia, Parit Raja, Batu Pahat 86400, Johor, Malaysia; 3 Research Unit for Robophilosophy & Integrative Social Robotics (RISR) Robophilosophy Lab, Aarhus University, 8000 Aarhus, Denmark; mustafa.abro@ieee.org; 4 Department of Defense and AI System Engineering, Sejong University, Gwangjin-gu, Seoul 05006, Republic of Korea; sufyanahmedali@sejong.ac.kr; 5 James Watt School of Engineering, University of Glasgow, Glasgow G12 8QQ, UK

**Keywords:** arch-shaped fabrication, energy harvesting, flexible electronics, self-powered sensors, triboelectric nanogenerator (TENG), sustainable energy

## Abstract

The growing adoption of flexible and portable electronic devices has created an increasing need for sustainable power sources that can operate without relying on conventional batteries. Triboelectric nanogenerators (TENGs) have attracted significant attention due to their ability to convert ambient mechanical energy into electrical energy. However, practical contact–separation TENGs may require additional spacers, elastic supports, or other structural components to maintain repeated contact and separation. This study presents a spacer-free arch-shaped TENG based on a laminated structure consisting of polytetrafluoroethylene (PTFE), paper, and aluminum. The arch configuration provides an inherent restoring tendency that facilitates contact–separation operation without a discrete spacer or additional elastic support. The fabricated device was experimentally evaluated through open-circuit voltage, short-circuit current, load-dependent electrical characterization, power measurement, capacitor charging, and LED illumination. Under the manual mechanical actuation conditions used in the present study, the device exhibited a maximum open-circuit voltage of 69 V, a reported peak short-circuit current of 68 μA, and a peak power output of 8.576 μW at an optimal load resistance of approximately 55 MΩ. Furthermore, the TENG charged a 4.7 µF capacitor to 7 V within approximately 60 s and directly illuminated 30 commercial light-emitting diodes (LEDs). These results demonstrate the feasibility of combining a simple spacer-free arch-shaped configuration with readily available materials for low-cost triboelectric energy harvesting. Long-term cyclic durability, controlled mechanical excitation, environmental stability, and matched structural control experiments remain important areas for future investigation.

## 1. Introduction

The rapid advancement of mobile and flexible electronic technologies has created a growing demand for lightweight, portable, and flexible power sources. Conventional power supplies, particularly batteries, can present limitations for such applications because of their finite lifetime, size, weight, and environmental considerations [1,2,3,4]. Consequently, researchers have increasingly explored alternative approaches for sustainable energy generation and self-powered electronic systems [5,6,7,8,9,10,11,12,13]. One of the most promising approaches in flexible electronics is the harvesting of ambient mechanical energy to generate electrical power [1,2,3,4]. Energy harvesting has emerged as an attractive alternative to conventional battery-based power systems. By converting naturally available environmental energy into usable electrical energy, energy harvesting technologies offer a sustainable and reliable means of powering electronic devices [14,15]. The ability to transform various forms of ambient energy into electricity has attracted considerable attention from researchers worldwide [16,17,18,19,20]. Among the available energy harvesting technologies, triboelectric nanogenerators (TENGs) have attracted considerable attention because they can convert ambient mechanical energy into electrical energy through triboelectrification and electrostatic induction [21,22,23,24,25]. TENGs harvest a wide range of mechanical motions and offer advantages, including lightweight construction, relatively simple fabrication, high output voltage, and compatibility with flexible materials [26,27,28]. These characteristics make TENGs attractive for wearable, portable, and self-powered electronic applications [26,27,28].

Despite these advantages, maintaining effective and repeatable contact–separation motion remains an important consideration in the design of practical contact–separation TENGs. Conventional configurations employ discrete spacers, elastic supports, multilayer structures, or other mechanical components to establish the required separation and restoring motion. Such additional components can increase structural complexity and may reduce the simplicity and accessibility of low-cost TENG architectures. Paper-based TENGs are particularly attractive because paper is inexpensive, lightweight, readily available, and compatible with straightforward fabrication. However, the structural configuration must still provide a suitable mechanism for repeated contact and separation between the triboelectric surfaces.

Previous studies have explored paper-based, folded, curved, and spacer-assisted TENG architectures using different triboelectric materials, surface modes, conductive-layer, structural configurations, and interface engineering [29,30,31]. These studies demonstrate that device geometry can be used to establish the mechanical motion required for triboelectric energy generation. However, these devices differ in their practical simplification of the mechanical contact–separation architecture, which remains an important design consideration. Moreover, the reported devices differ in material composition, design fabrication, dimensions, excitation conditions, and measurement procedures, making direct performance comparisons difficult. In the existing paper-based contact–separation TENGs, auxiliary spacers, elastic layers, and other restoring components were employed to manage the repeated separation of the device surfaces, which increases the number of components and assembly requirements. Therefore, there remains practical interest in simple architectures in which the restoring function required for contact–separation operation is incorporated into the device structure while maintaining readily accessible materials and uncomplicated fabrication.

Recent studies have further expanded TENG technologies toward self-powered sensing, environmental monitoring, and energy harvesting from diverse mechanical and liquid sources. TENG-based systems have been demonstrated for rapid environmental contaminant detection and integrated water monitoring and treatment [32], while other studies have explored liquid-based triboelectric systems for sustainable energy harvesting and long-term sensing applications [33,34,35]. Triboelectric sensing has also been integrated with portable and robotic platforms for on-site chemical detection [36]. These developments highlight the broader potential of TENGs as simple and self-powered platforms for energy harvesting and sensing applications.

Recent advances have further extended triboelectric technologies toward high-output energy harvesting, flexible and wearable devices, durable self-powered systems, intelligent sensing, and noncontact perception [37,38,39,40,41,42]. These studies demonstrate the potential of triboelectric platforms for combining mechanical-energy harvesting with sensing, human–machine interaction, and autonomous electronic applications. At the same time, improving electrical output, mechanical durability, and reliable operation remains important for practical deployment. These developments highlight the continuing need for simple and accessible TENG architectures that can provide useful electrical output while maintaining straightforward fabrication.

In this work, a laminated arch-shaped TENG consisting of paper, PTFE, and aluminum is fabricated and experimentally characterized as a spacer-free energy harvesting architecture. The arch configuration provides an inherent restoring tendency that facilitates contact–separation operation without a discrete spacer or additional elastic restoring component. This study focuses on fabrication, electrical characterization, load-dependent power evaluation, capacitor charging, and LED illumination. The objective is to demonstrate the feasibility of combining a simple arch-shaped structural configuration with readily available materials for low-cost triboelectric energy harvesting. The contribution of this work is the experimental demonstration of a simple spacer-free arch-shaped TENG fabricated from readily available paper, PTFE, and aluminum. Rather than considering the arch geometry itself as a new TENG concept, this study investigates its use as an integrated restoring structure within a simple laminated configuration. The electrical characterization and practical demonstrations provide an initial assessment of the feasibility of this architecture for low-cost triboelectric energy harvesting.

## 2. Principal of Triboelectric Nanogenerator

### 2.1. Triboelectrification Effect

As human civilization evolves, the comprehension of static electricity has significantly advanced, leading to a more sophisticated understanding of its generation mechanisms. A well-known explanation for this occurrence is referred to as triboelectricity. When the surfaces of two distinct materials interact, a specific quantity of charge is exchanged at their contact point [43]. The transfer of charge typically occurs through the thermodynamic movement of electrons from elevated energy states in the valence band of one material to vacant lower-energy states in the conduction band of another. The contact potential difference (CPD) is a critical factor governing the efficiency and kinetics of this interfacial electron transfer. The distribution of energy and density of states on both surfaces correlates with the CPD [44]. Moreover, the alignment between the Fermi level of the conductor and the effective Fermi level of the insulator critically influences the magnitude of the resulting CPD [45]. A critical parameter for understanding electronic properties in insulating materials is the effective Fermi level, which quantifies the energy separation between acceptor and donor states. It provides fundamental insights into charge carrier distribution, concentration, and transport mechanisms. Understanding this separation is fundamental to the study of electronic band structures and the behavior of defects within insulating materials [46]. In the context of conductor–insulator interactions and the interfaces between different insulating materials, any charge that is transferred to the insulating surface becomes spatially confined and concentrated in that region, rather than dissipating instantaneously. This phenomenon underscores the significance of charge localization effects at these interfaces, which can have implications for the electrical behavior and dielectric properties of the materials involved. The polarity of the charge retained on an insulating surface after contact is determined by the direction of electron flow, which is governed by the CPD. This static charge accumulation is known as contact charging. When the materials slide horizontally, the dynamic variation in this contact charge is termed the triboelectric effect [47].

### 2.2. Triboelectric Series

After conducting nearly a decade of rigorous investigation into TENG, it has been verified that frictional electrification occurs across a diverse array of materials commonly encountered in human interactions. This phenomenon underscores the significance of material properties in the context of energy harvesting and has broad implications for the development of TENG technologies. Consequently, the choice of friction layers for applications involving TENG has become more diverse and varied [48]. The output signal changes considerably depending on the materials used for the friction layer. Research indicates that the capacity of various materials to acquire and relinquish electrons significantly influences the performance of TENGs, as this phenomenon is intrinsically linked to the materials’ relative polarity. A systematic assessment of the relative polarity of materials enables their organization into a triboelectric series, which ranks them by their tendency to acquire a positive or negative charge upon contact [49]. The disparities in relative polarity between two distinct materials significantly influence the magnitude of the triboelectric effect and the amount of charge that is transferred during this interaction. Studies have resulted in the creation of a triboelectric series that ranks materials based on the charges they accumulate on their surfaces. Materials that can achieve the highest CPD have been recognized, and efforts continue to develop more precise triboelectric sequences for these substances. The structure is versatile enough to incorporate various functional groups, enabling highly customizable dielectric characteristics and surface polarization. A solid comprehension of the mechanisms driving electron transfer has been developed, including material properties, the connection between transferred charges, and environmental elements such as temperature and external electric fields. Nevertheless, the more dynamic components of triboelectrification and its fundamental mechanisms still need further investigation.

### 2.3. Maxwell’s Displacement Current

The pioneering concept of the nanogenerator (NG) was introduced in 2006 by Zhong Lin Wang’s research group. This groundbreaking work established the foundation for nanoscale energy harvesting by demonstrating the conversion of mechanical energy into electricity using nanomaterials and structures [14]. The TENG, or triboelectric nanogenerator, was first identified in 2012, leading to a series of comprehensive studies aimed at exploring the underlying principles of nanogenerator technology. As research progressed, the theoretical framework surrounding this field has been progressively refined. Among the most noteworthy findings is the identification of Maxwell’s displacement current as a fundamental theoretical source that underpins the operation of nanogenerators. This insight has substantial implications for the development and optimization of energy harvesting technologies. Gauss’s Law states that an electric flux density *D* diverging at a point has the free charge density $\rho_{f}$ at that point, as expressed in Equation (1).

(1) $$\nabla \cdot D=\rho_{f},$$

where ∇ denotes the divergence operator. However, the divergence of the magnetic flux density B is zero everywhere in the field, as per the definition of Gauss’s magnetic law expressed in Equation (2).

(2) ∇·B=0

From Faraday’s law, the time-varying magnetic field induces an electric field *E* in the closed space. We have

(3) $$\nabla \times E=-\frac{\partial B}{\partial t}$$

The magnetic fields *H* are the addition of free charge density $J_{f}$ and time-varying electric fields. From Maxwell Ampere’s law, we know that

(4) $$\nabla \times H=J_{f}+\frac{\partial D}{\partial t},$$

The electric field density *D* is related to the polarization field density by

(5) $$D=\epsilon_{0}E+P,$$

where $\epsilon_{0}$ represents the permittivity dielectric constant. The polarization field density in the isotropic media is defined $P=(\epsilon -\epsilon_{0})E$, and thus, the electric field density becomes D=ϵE. In Equation (4), $\frac{\partial D}{\partial t}$ is known as the Maxwell displacement current, which is further expressed by

(6) $$J_{D}=\frac{\partial D}{\partial t}=\varepsilon_{0}\frac{\partial E}{\partial t}+\frac{\partial P}{\partial t},$$

The displacement current represents a phenomenon distinct from the conventional current associated with the flow of free electrons. It arises from the presence of a time-varying electric field, whether in a vacuum or within a material medium. This phenomenon is notably influenced by the microscopic, time-dependent movements of bound charges within atoms and the dielectric polarization occurring in the material. In Equation (5), considering isotropic media, the two terms are unified, and the displacement current is now expressed by $J_{D}=\epsilon \frac{\partial E}{\partial t}$. The displacement current in triboelectric materials arises from the polarization density induced by static charges, as defined in Equation (7). This relationship exemplifies the fundamental coupling between electrostatic phenomena and the material’s dielectric response.

(7) $$J_{D}=\frac{\partial D}{\partial t}=\varepsilon \frac{\partial E}{\partial t}+\frac{\partial P_{s}}{\partial t},$$

The concept of displacement current forms a foundational element for understanding electromagnetic waves. Furthermore, it serves as a critical theoretical framework and source for the operation of nanogenerators. This indicates that the current generated within these systems is attributed to the static charge present on the surface of the polarization field [50].

## 3. Applications of Triboelectric Nanogenerator

The operation of TENGs is categorized into four fundamental modes: vertical contact–separation, lateral sliding, single-electrode, and freestanding triboelectric-layer. The existence of these varied operational mechanisms underscores the adaptability of TENGs for diverse mechanical energy harvesting scenarios. Each mode employs distinct mechanisms of triboelectric effects and contact electrification to capture mechanical energy, enhancing the versatility and effectiveness of TENGs in energy harvesting applications [51]. Table 1 offers an overview of the latest studies on triboelectric nanogenerators (TENGs) within robotic hands, highlighting the operational modes employed in each research effort.

An analysis of the literature, summarized in Table 1, indicates a prevalence of vertical contact–separation and single-electrode modes in robotic hands, attributed to their straightforward design and substantial energy output. The freestanding and lateral sliding modes, while less common, offer complementary benefits for specific scenarios. The distinct advantages and limitations of each mode (Table 2) imply that the development of an optimal TENG for a given application likely involves the strategic integration of these fundamental operational models.

The paper-based triboelectric nanogenerators documented in the literature implement discrete spacers, elastic supports, polymers and multi-stack designs to maintain contact–separation and restoring force, such as discussed in [43,48,53,54,55]. In contrast to these traditional flat, stacked, or spacer-based TENGs, the proposed arch-shaped laminated design functions as an inherent spring, facilitating efficient and consistent contact–separation motion without using external spacers or complex fabrication process. The arch configuration provides an inherent restoring tendency that facilitates contact–separation motion without requiring a discrete external spacer. This structural advancement is noteworthy and has not been widely implemented in paper-based TENGs. Many high-performance triboelectric nanogenerators typically rely on costly nanomaterials, surface functionalization, or intricate composites. In contrast, the proposed design method achieves competitive outputs of 69 V, 68 μA, and 8.576 μW using only standard, unmodified office paper, PTFE tape, and aluminum foil. The measured results demonstrate that useful electrical output can be obtained using readily available paper, PTFE, and aluminum without advanced material processing. The proposed device efficiently powered 30 LEDs and charged a 4.7 μF capacitor to 7 V in just 60 s, demonstrating its suitability for low-power electronics and IoT applications. The fabrication process uses readily available materials and straightforward assembly steps, which may facilitate low-cost prototyping.

## 4. Materials, Fabrication, and Experimental Methods

The arch-shaped configuration was selected to provide an inherent restoring tendency for contact–separation operation without requiring a discrete external spacer. The device was fabricated using readily available paper, PTFE, and aluminum, with the layer dimensions and thicknesses defined according to the fabrication procedure described below.

### 4.1. Fabrication of the Arch-Shaped TENG

**Figure 1:**
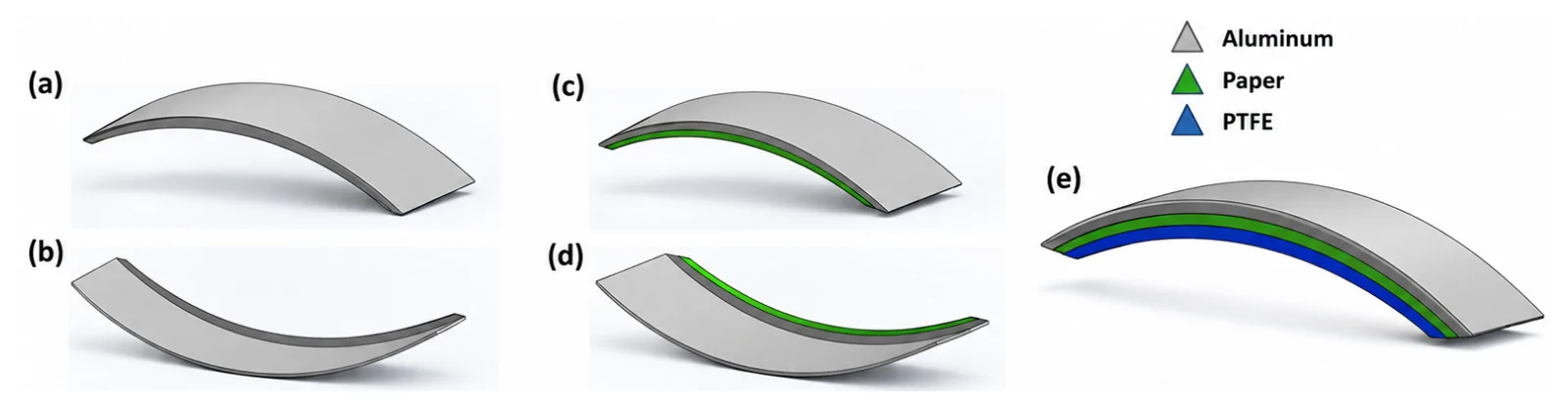
Complete fabrication process of the fabrication of the triboelectric nanogenerator, layer by layer: (**a**,**b**) upper and lower layer of the TENG made up of thin film of aluminum, (**c**,**d**) aluminum pasted over the paper, (**e**) three layers of the aluminum, paper and PTFE.

The fabricated TENG has a five-layer structure: two positive layers, one negative layer, and two electrodes. The positive triboelectric layer consists of paper (0.25 mm thick) cut to 4 cm × 1.8 cm (Figure 1a). The negative layer is a PTFE film of the same dimensions (4 cm × 1.8 cm) but with a thickness of 0.3 mm (Figure 1b). Both electrodes are made from aluminum tape (0.8 mm thick, 4 cm × 1.8 cm), as shown in Figure 1c,d. All layers were cut to shape using a Sesser.

### 4.2. Assembling PTFE, Paper and Aluminum for Harvesting Energy

**Figure 2:**
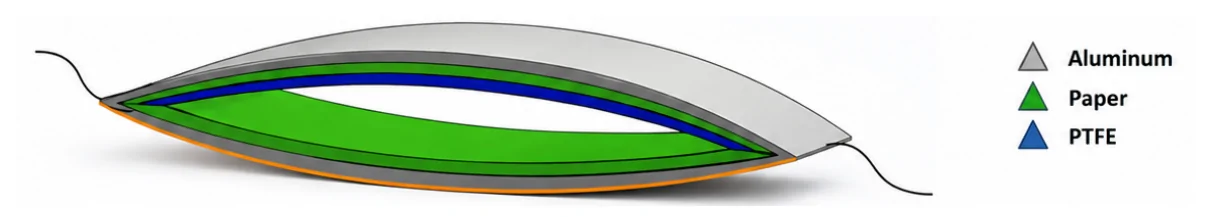
Design and structure of the fabricated nanogenerator made up of PTFE, paper, aluminum and two copper wires for the flow of charges.

The five-layer structure was assembled as two laminated sections arranged to form the arch-shaped configuration. One section consisted of three layers comprising aluminum, paper, and PTFE, while the other section consisted of two layers comprising aluminum and paper. The aluminum layers served as the electrodes and charge collectors, and conductive wires were connected to the aluminum electrodes for electrical measurement. The two sections were arranged face-to-face in an arch configuration with an air gap between them, as illustrated in Figure 2. The corners of the sections were joined using transparent adhesive tape to maintain the assembled arch configuration. This arrangement enables the paper and PTFE triboelectric surfaces to approach and separate during external mechanical actuation without requiring a separate discrete spacer.

### 4.3. Working Mechanism

**Figure 3:**
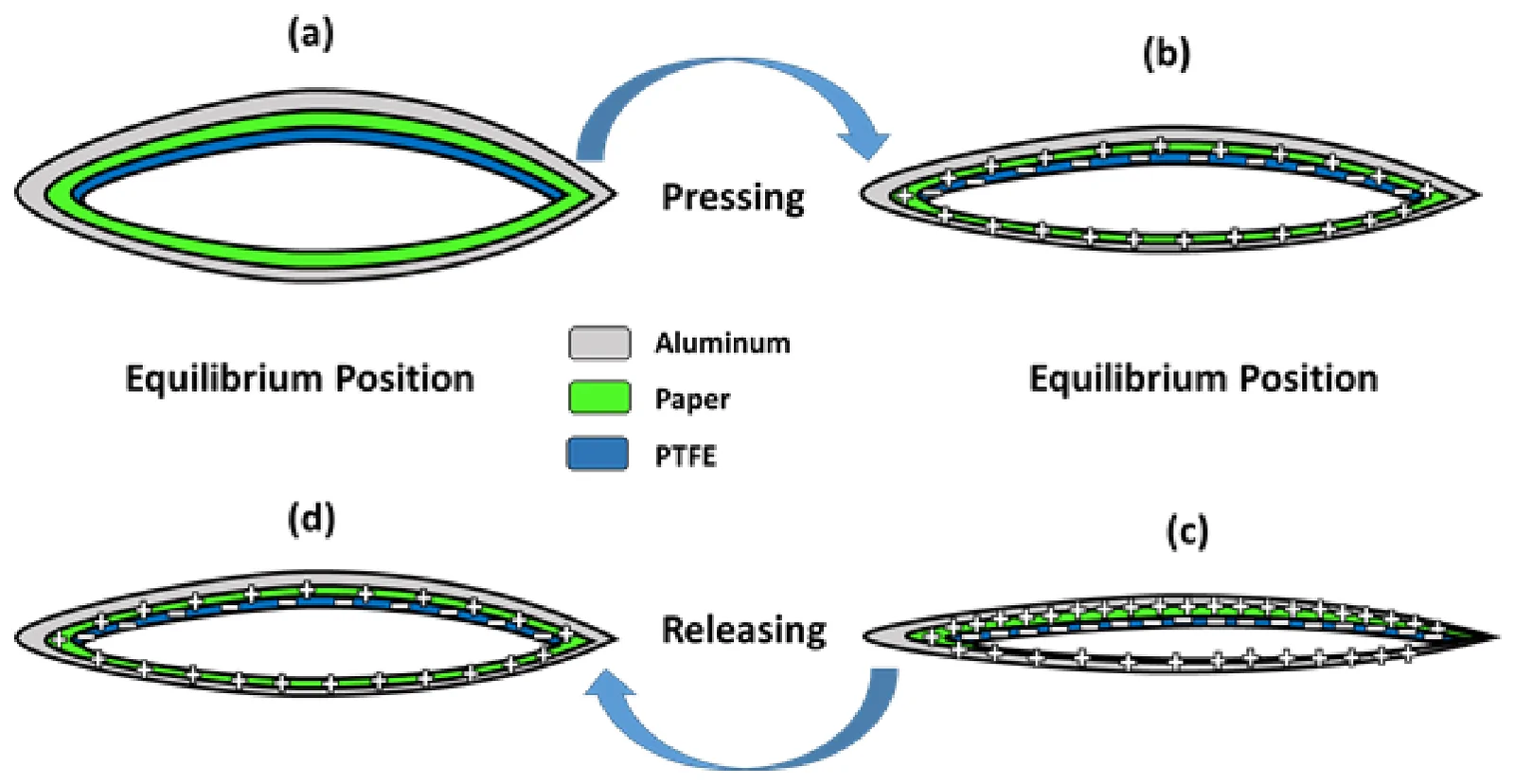
Working mechanism of fabricated arch-shaped TENG: (**a**) the fabricated device at the initial position/shape, (**b**) when the fabricated device is pressed, (**c**) when the fabricated device is fully pressed (when paper and PTFE come into contact with each other), (**d**) when the fabricated device is released to return to its original state/shape.

The TENG operates according to the contact–separation mode. In the initial arch configuration, the paper and PTFE surfaces are separated by the air gap, as illustrated in Figure 3a. When an external mechanical force is manually applied, the arch is deformed and the triboelectric surfaces are brought into contact, as shown in Figure 3b,c. Contact between the paper and PTFE produces triboelectric charge separation at their interfaces. When the external force is released, the restoring tendency of the arch configuration causes the layers to separate, producing a change in the electrostatic potential and driving charge through the external circuit, as illustrated in Figure 3d. Repeated pressing and releasing therefore produces an alternating electrical output.

### 4.4. Electrical Characterization

The electrical output of the fabricated TENG was characterized under repeated manual mechanical actuation. Open-circuit voltage (V_oc_) and short-circuit current (I_sc_) were recorded to evaluate the electrical response generated during contact–separation operation. For load-dependent characterization, external resistances ranging from 1 MΩ to 105 MΩ were connected to the TENG, and the corresponding output voltage and current were measured. For the practical energy-storage demonstration, the alternating-current output of the TENG was connected to a DB107 full-wave bridge rectifier, and the rectified output was used to charge a 4.7 μF capacitor. The capacitor voltage was monitored during charging using an oscilloscope. The rectified output was also used to demonstrate the direct illumination of 30 commercial LEDs.

## 5. Results and Discussion

The fabricated arch-shaped TENG was assembled and maintained in the intended arch configuration using transparent adhesive tape. The electrical performance of the device was characterized under repeated manual mechanical actuation. Because the original experimental campaign did not include calibrated measurements of applied force, displacement, loading speed, or excitation frequency, the reported electrical results represent the response obtained under the manual actuation conditions used during the original proof-of-concept experiments rather than standardized performance under a prescribed mechanical input. This methodological approach facilitated a comprehensive analysis of the device’s operational efficacy and longevity.

**Figure 4:**
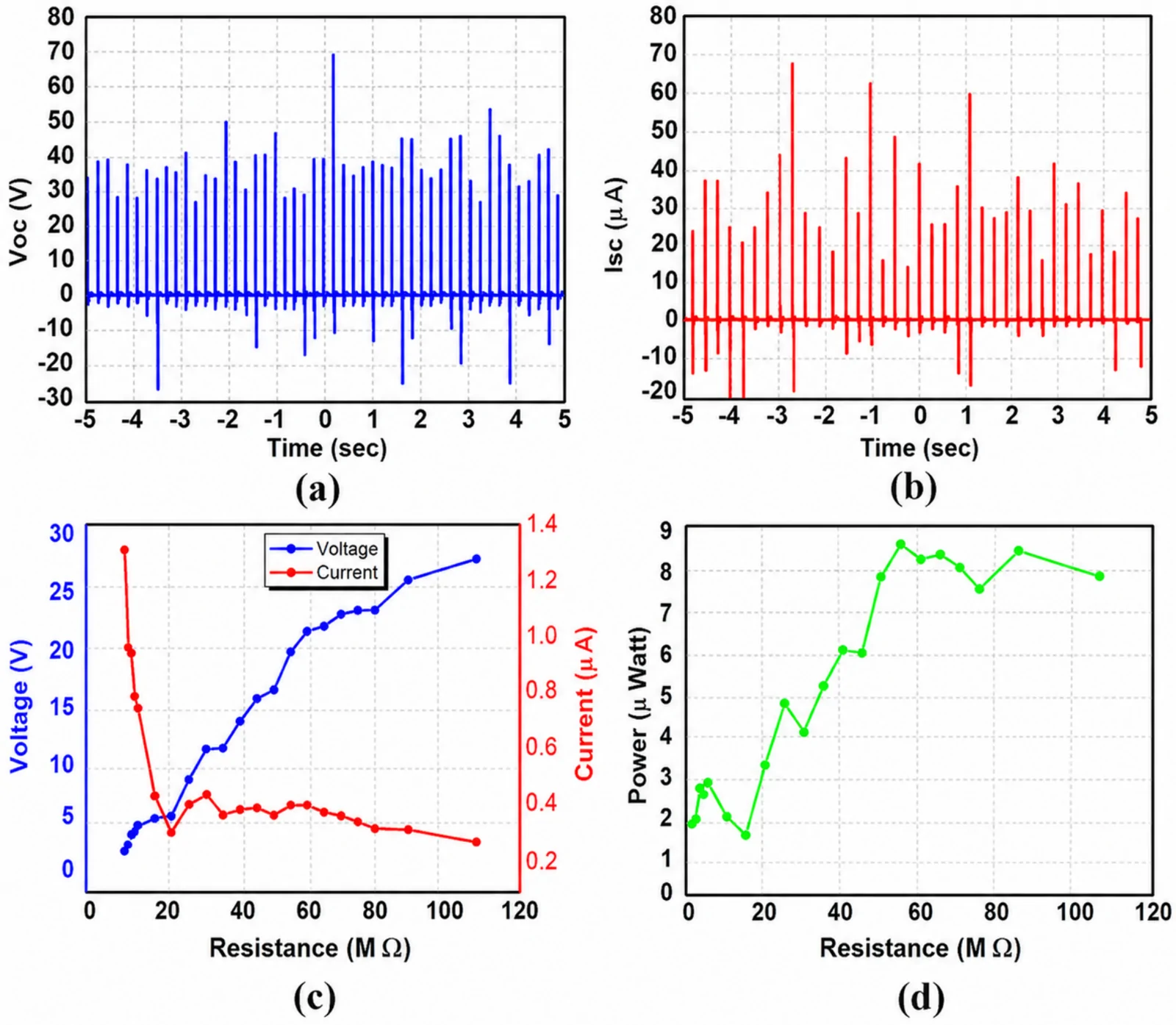
(**a**) Open-circuit voltage ($V_{\mathrm{oc}}$) of the fabricated TENG, (**b**) short-circuit current ($I_{\mathrm{sc}}$) of the fabricated TENG, (**c**) voltage and current at the different loads, and (**d**) maximum power achieved 8.576 μW at optimal load of 55 MΩ.

The electrical output of the fabricated TENG was characterized through measurements of open-circuit voltage and short-circuit current. The TENG produced an alternating V_oc_ signal with a maximum positive amplitude of 69 V and a negative amplitude of 28 V, as depicted in Figure 4a. The corresponding $I_{\mathrm{sc}}$ exhibited a peak value of 68 μA (Figure 4b), which is reported here as the peak short-circuit current rather than an average or RMS current. To evaluate the device performance under practical loading conditions, the proposed TENG device was characterized using external load resistances varying from 1 MΩ to 105 MΩ. As shown in Figure 4c, the loaded output voltage demonstrated an increase with increase in resistance, achieving a maximum voltage of 29 V at 105 MΩ, while the output current exhibited a corresponding decrease, with a peak of 1.3 μA recorded at the lowest load-resistance. The output power across the diverse range of resistances was calculated, resulting in a maximum output power of 8.576 μW at an optimal load resistance of ≈55 MΩ, corresponding to a loaded output voltage of approximately 21.7 V (Figure 4c,d). This observed behavior aligns with fundamental circuit principles and the dynamics of ohmic loss mechanisms. For contextual comparison, the electrical performance of the proposed TENG is compared with select low-cost and paper-based TENGs reported in the literature, as summarized in Table 3. We note that Reference [62] presented a maximum power density of 2.1 μW/cm^2^, which is not directly comparable with the 8.58 μW total power of the proposed device, because the two quantities denote different evaluation metrics. Because the reported devices were evaluated using different materials, dimensions, structural configurations, excitation conditions, and measurement procedures, the values should be interpreted as contextual comparisons rather than a direct performance ranking.

**Figure 5:**
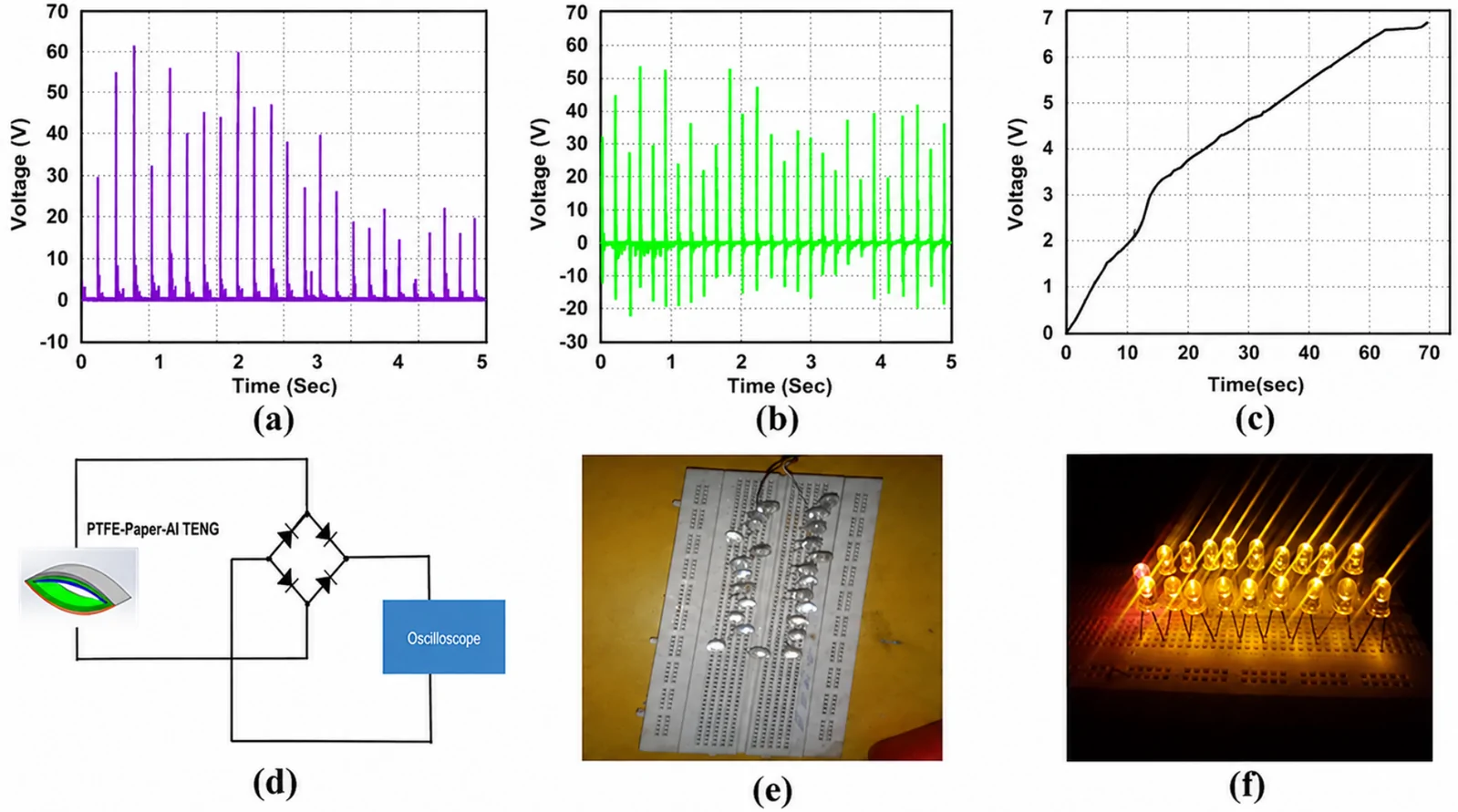
(**a**) Representative open-circuit voltage (V_oc_) waveform of the fabricated TENG under continuous manual mechanical excitation, (**b**) rectified output-voltage waveform after the full-wave bridge rectifier, (**c**) capacitor-charging profile of the 4.7 μF capacitor using the rectified output, (**d**) circuit diagram of the rectifier attached to TENG and oscilloscope, (**e**) 30 LEDs before powering, (**f**) 30 LEDs powered up by the fabricated TENG.

Figure 5a presents a representative open-circuit voltage ($V_{\mathrm{oc}}$) waveform of the fabricated TENG under continuous manual actuation, showing the repeated voltage generation across consecutive contact–separation cycles. A DB107 full-wave bridge rectifier was used to rectify the fabricated device alternating-current (AC) output voltage, showing a peak voltage ≈50 V, as shown in Figure 5b, which is subsequently utilized to charge a 4.7 μF capacitor at 7 V in ≈60 s (with five trials), highlighting the repeatable capacitor-charging behavior observed under the tested manual actuation conditions, as illustrated in Figure 5c. Figure 5d demonstrates the experimental scenario used for device rectification and performance measurement. The arch-shaped TENG was connected to a full-wave bridge rectifier, whereas its rectified output voltage was monitored using an oscilloscope. Furthermore, the TENG effectively and consistently powered thirty commercial LEDs across multiple activations, which illuminated immediately upon mechanical activation (Figure 5f) and turned off when the force was released (Figure 5e), providing a clear visual indication of its self-powering capability.

The findings demonstrate the feasibility of the arch-shaped TENG as a simple and low-cost energy harvesting device under the tested conditions. This device is well-suited for the sustainable powering of low-power electronic devices and holds significant promise for applications in the realms of wearable and portable electronics.

The literature comparison in Table 3 provides context for the reported electrical output of the proposed device. The proposed configuration uses paper, PTFE, and aluminum with a simple laminated arch-shaped structure and does not require advanced material functionalization. Nevertheless, differences in device geometry, active dimensions, excitation conditions, and measurement procedures prevent a direct quantitative ranking of the reported devices. The results therefore demonstrate the feasibility of obtaining useful electrical output from the proposed low-cost configuration rather than establishing superiority over previously reported TENG architectures. Specifically, the device’s high short-circuit current relative to other bio-derived or waste-based TENGs underscores its superior capability for directly powering electronic components and rapidly charging storage elements.

## 6. Conclusions

This study experimentally demonstrated a spacer-free arch-shaped triboelectric nanogenerator fabricated using readily available paper, PTFE, and aluminum. The arch configuration provides an inherent restoring tendency that facilitates contact–separation operation without a discrete external spacer. Under the manual mechanical actuation conditions used in the original experimental campaign, the device produced a maximum open-circuit voltage of 69 V and a maximum transient short-circuit current of 68 μA. Load-dependent characterization showed a maximum output power of 8.576 μW at an optimal load resistance of approximately 55 MΩ, corresponding to a loaded voltage of approximately 21.7 V. The generated electrical output was further demonstrated through the charging of a 4.7 μF capacitor to 7 V in approximately 60 s and the illumination of 30 commercial LEDs.

The results demonstrate the feasibility of using a simple laminated arch-shaped configuration and readily available materials for low-cost triboelectric energy harvesting. However, the present study is limited to proof-of-concept electrical characterization under manual mechanical actuation. The applied force, displacement, loading speed, and excitation frequency were not quantitatively controlled or recorded, and a matched flat control structure was not experimentally evaluated. In addition, long-term cyclic durability and the effects of temperature and relative humidity on electrical performance were not systematically investigated. These limitations prevent conclusions regarding long-term reliability or environmental stability.

Future work should therefore investigate controlled mechanical excitation, matched structural comparisons, long-term cyclic operation, and environmental conditioning under different temperature and relative-humidity levels. Such investigations would provide quantitative evidence of performance retention, degradation behavior, and environmental sensitivity and would further clarify the suitability of the proposed architecture for practical energy harvesting applications.

## Figures and Tables

**Table 1:** Applications of various modes of TENG.

Ref.	Work Mode	Advantages
[52]	Vertical Contact–Separation Mode	The all-fiber-based TENG that has been engineered demonstrates remarkable flexibility and retains consistent sheet resistance despite considerable mechanical strain caused by folding.
[53]	Single-Electrode Mode	The system demonstrates considerable flexibility, robust compatibility, and high precision in electrical signal recognition.
[54]	Vertical Contact–Separation Mode	A sensitivity of 2 V/N was achieved by the device functioning as a contact–separation-mode TENG, underscoring its efficient conversion of mechanical force into an electrical output.
[55]	Vertical Contact–Seaeration Mode	The device possesses a dual nature, characterized by both flexibility and durability, which significantly enhances its capacity to sense a variety of environmental conditions. It is adept at detecting both static and dynamic pressure variations within the external milieu, thereby augmenting its functionality across a spectrum of diverse applications. This capability underscores its potential utility in various fields where precise environmental monitoring is essential.
[56]	Freestanding Triboelectric-Layer Mode	The device operates under a secure, high-static-voltage condition, ensuring that it poses no hazards to researchers. Moreover, it is designed as a self-sustaining system that leverages TENG technology, eliminating the necessity for external power sources.
[57]	Single-Electrode Mode	The device exhibits a significant capability for energy harvesting via hand clapping, achieving a maximum open-circuit voltage of up to 320 V for the GCP-TENG (Guide Coupled Piezoelectric Triboelectric Nanogenerator). This innovative method underscores the potential of harnessing ambient mechanical energy sources for sustainable power generation.
[58]	Single-Electrode Mode	The devices under discussion are distinguished by their inherent simplicity, reliability, utility, and efficiency. Moreover, the associated processes have been demonstrated to be cost-effective and are particularly well-suited for applications in industrial manufacturing contexts.
[59]	Freestanding Triboelectric-Layer Mode	The gripper demonstrates a remarkable capacity for non-destructive grasping, coupled with the ability to execute accurate dimensional measurements and precise sorting of objects. Significantly, the system maintains an exceptionally low measurement error rate of 2.22%, which underscores its reliability and accuracy in operational performance. This level of precision is vital for applications requiring high fidelity in handling and categorizing materials.
[60]	Vertical Contact–Separation Mode	The incorporation of plasticized PVC-gel presents a methodology capable of producing substantial output power, facilitating the illumination of numerous LEDs with minimal tactile engagement. This approach underscores the potential for efficient energy transfer and activation in electronic applications.

**Table 2:** Key characteristics, advantages, and limitations of primary TENG operational modes [61].

Operational Mode of TENG	Key Features	Challenges
Vertical Contact–Separation Mode	The structure is characterized by its simplicity, facilitating ease of fabrication. It demonstrates the capability for instantaneous high output, making it particularly advantageous for multilayer integration applications.	Friction materials exhibit a susceptibility to wear under conditions of repetitive stress and interaction, highlighting their limitations in durability and performance over time.
Lateral Sliding Mode	The energy conversion efficiency is notably elevated, characterized by a variety of forms that facilitate seamless integration and packaging. This versatility contributes to its extensive applicability across diverse fields.	The device takes up a significant amount of space.
Single-Electrode Mode	The material exhibits lightweight properties, exceptional wear resistance, and a high degree of flexibility during operational conditions, thereby rendering it highly suitable for applications involving autonomous sensor technologies.	The suboptimal efficiency inherent in energy collection processes represents a substantial challenge within the domain of energy management and sustainable practices. This inefficiency poses critical implications for the overall effectiveness of energy systems and their capacity to meet both current and future energy demands while minimizing environmental impacts.
Freestanding Triboelectric-Layer Mode	The characteristics of diverse mechanisms, enhanced durability, and increased current capacity present a notable distinction when compared to the single-electrode mode.	Fluctuating energy signal dynamics.

**Table 3:** Performance comparison with other low-cost TENGs.

Ref.	Triboelectric Layers & Electrodes	Key Structural Feature	Maximum Total Power	Open-Circuit Voltage, V_oc_	Short-Circuit Current, I_sc_	Key Application
[48]	Graphite/Paper	Single-electrode mode on paper	4.2 μW	320 V	-	Hand clapping energy harvesting
[62]	PVDF-Paper/Natural Rubber/Cu	Flat, vertical contact–separation	–	33 V	3 μA	Charging a 10 μF capacitor to 2.6 V in ∼120 s
[63]	Al/PTFE-Si-Rubber on Paper	Flat, foldable sandwich structure	–	153 V (folded)	2.2 μA (folded)	Powering a wristwatch and LEDs
[64]	LDPE/Oxidized LDPE/Al (carton)	Sandwich structure from recycled packaging	–	200 V	0.4 μA	Charging 10 μF to 3.5 V in ∼4 min; Lighting LEDs
Proposed method	Paper/PTFE/Al	Monolithic arch-shape (spacer-free)	8.58 μW (at 55 MΩ load)	69 V	68 μA	Charging 4.7 μF to 7 V in ≈60 s; powering 30 LEDs

Note: Only directly comparable performance metrics are included.

## Data Availability

The data presented in this study are available on request from the corresponding author.
